# Oscillatory infrasonic modulation of the cochlear amplifier by selective attention

**DOI:** 10.1371/journal.pone.0208939

**Published:** 2019-01-07

**Authors:** Constantino D. Dragicevic, Bruno Marcenaro, Marcela Navarrete, Luis Robles, Paul H. Delano

**Affiliations:** 1 Neuroscience Department, Facultad de Medicina, Universidad de Chile, Santiago, Chile; 2 Programa de Fisiología y Biofísica, ICBM, Facultad de Medicina, Universidad de Chile, Santiago, Chile; 3 Otolaryngology Department, Clinical Hospital, Universidad de Chile, Santiago, Chile; 4 Biomedical Neuroscience Institute, Universidad de Chile, Santiago, Chile; Universidad de Salamanca, SPAIN

## Abstract

Evidence shows that selective attention to visual stimuli modulates the gain of cochlear responses, probably through auditory-cortex descending pathways. At the cerebral cortex level, amplitude and phase changes of neural oscillations have been proposed as a correlate of selective attention. However, whether sensory receptors are also influenced by the oscillatory network during attention tasks remains unknown. Here, we searched for oscillatory attention-related activity at the cochlear receptor level in humans. We used an alternating visual/auditory selective attention task and measured electroencephalographic activity simultaneously to distortion product otoacoustic emissions (a measure of cochlear receptor-cell activity). In order to search for cochlear oscillatory activity, the otoacoustic emission signal, was included as an additional channel in the electroencephalogram analyses. This method allowed us to evaluate dynamic changes in cochlear oscillations within the same range of frequencies (1–35 Hz) in which cognitive effects are commonly observed in electroencephalogram works. We found the presence of low frequency (<10 Hz) brain and cochlear amplifier oscillations during selective attention to visual and auditory stimuli. Notably, switching between auditory and visual attention modulates the amplitude and the temporal order of brain and inner ear oscillations. These results extend the role of the oscillatory activity network during cognition in neural systems to the receptor level.

## Introduction

In natural environments animals are surrounded by a variety of sensory stimuli. As the nervous system has a limited capacity for processing all sensory stimuli, individuals require of attention to focus their cognitive resources on the most relevant. Selective attention is a top-down form of attention in which one sensory modality is important to accomplish a given task and the other modalities are irrelevant or even distracting [1]. At the mechanistic level, it has been proposed that selective attention can function as a biological filter, meaning that neural responses to the attended stimulus are enhanced, while responses to unattended stimuli can be diminished. Whether these processes occur at the central nervous system only, or also at more peripheral levels has remained controversial for many years [2–4]. In the case of selective attention to visual stimuli with auditory distractors, modulation of auditory response gains is clearly observed at the cortical level [3,5], while at the peripheral level, conflicting results have been reported, including positive [6–8] and negative findings [3,4,9]. Although the gain control of sensory responses by attention is probably the principal mechanism of attentional selection, this process does not explain all of the neural modulations observed during attention, since the nervous system could use additional mechanisms for the selection of a relevant stimulus [5–8].

The oscillatory nature of the nervous system has been suggested as a general mechanism for perception and attention in vertebrates and invertebrates [10–13]. Amplitude and phase changes of brain oscillations in specific frequency bands have been proposed as mechanisms of attentional selection [13–15], which could allow local or large scale synchronization among different brain areas [10,16]. However, whether cortical oscillations modulate cochlear responses at the receptor level during selective attention to visual stimuli is unknown.

Here, we used an alternating visual/auditory selective attention task in humans (based on [17]) and measured electroencephalographic (EEG) activity simultaneously to a virtual channel of the amplitude of distortion product otoacoustic emissions (DPOAE) that allowed us to examine in the frequency domain, the single-trial dynamics between cortical electrical oscillations and hypothetical oscillatory activity of the cochlear amplifier [18,19].

## Results

**Fig 1:**
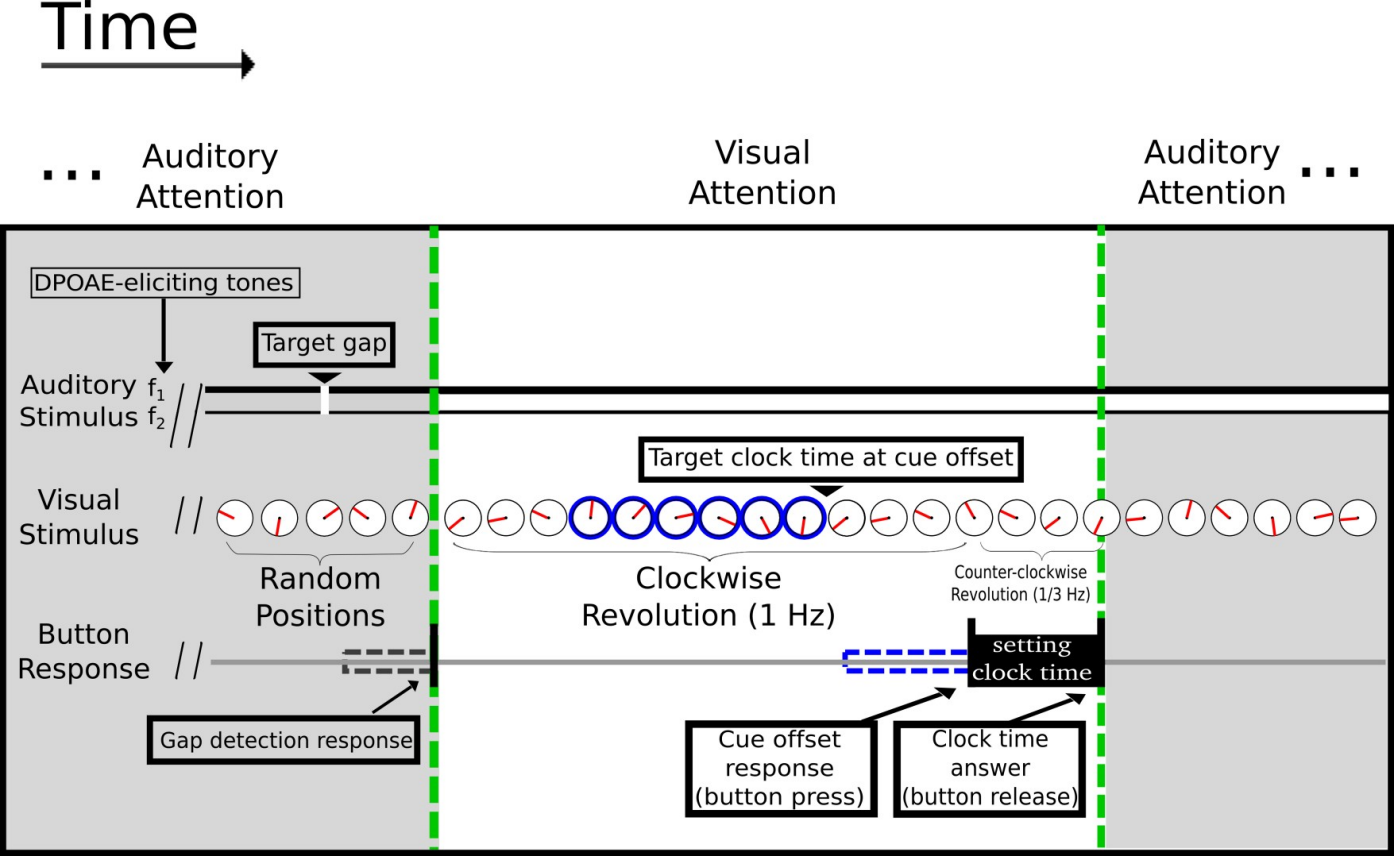
Illustration of the visual/auditory selective attention tasks. Subjects were required to alternate their attention between the visual and auditory modalities after each trial. The green dashed lines delimit the switch between auditory (grey shaded area) and visual (white area) attention. During visual attention a minute indicator (shown in red) rotates clock-wise at 1 Hz. After a random passive period of 2,000–2,500 ms, a peripheral clock rim appears as a temporal visual cue (shown in blue) and remains on for a variable period of 1,500–2,500 ms. Subjects were asked to report the position of the clock at the off-set of the peripheral clock rim. Simultaneously, in order to evoke DPOAEs, two tones (f1 and f2) were presented continuously, without silence gaps, during the visual attention task. On selective auditory attention trials (shaded grey), volunteers were required to report a brief (2–4 ms) silence gap embedded in the continuous DPOAE-eliciting tones. Gap detection triggered the switch to the random passive period of 2,000–2,500 ms before the initiation of the visual attention task.

Continuous 32-channel EEG and DPOAE signals were recorded simultaneously in 14 subjects performing alternating tasks that required attentional switches between visual and auditory perceptual modalities (Fig 1). Both modalities required high temporal acuity in detecting time in a revolving clock (visual) or a brief gap of silence embedded in continuous DPOAE-eliciting pairs of tones (auditory). Time and frequency averaged EEG and DPOAE signals were analyzed in a period of delimited high expectancy (selective attention) for both modalities, corresponding to the period before the appearance of auditory and visual targets (from 0 ms to 1500 ms) and were compared with the previous period (-1500 ms to 0 ms). In order to analyze EEG and DPOAE signals as part of the same functional network during selective attention, we added the DPOAE amplitude as an additional channel in EEG analyses. The DPOAE signal was evaluated using the amplitude of the frequency band surrounding (± 50 Hz) the 2f1-f2 component (S1 and S2 Figs).

**Fig 2:**
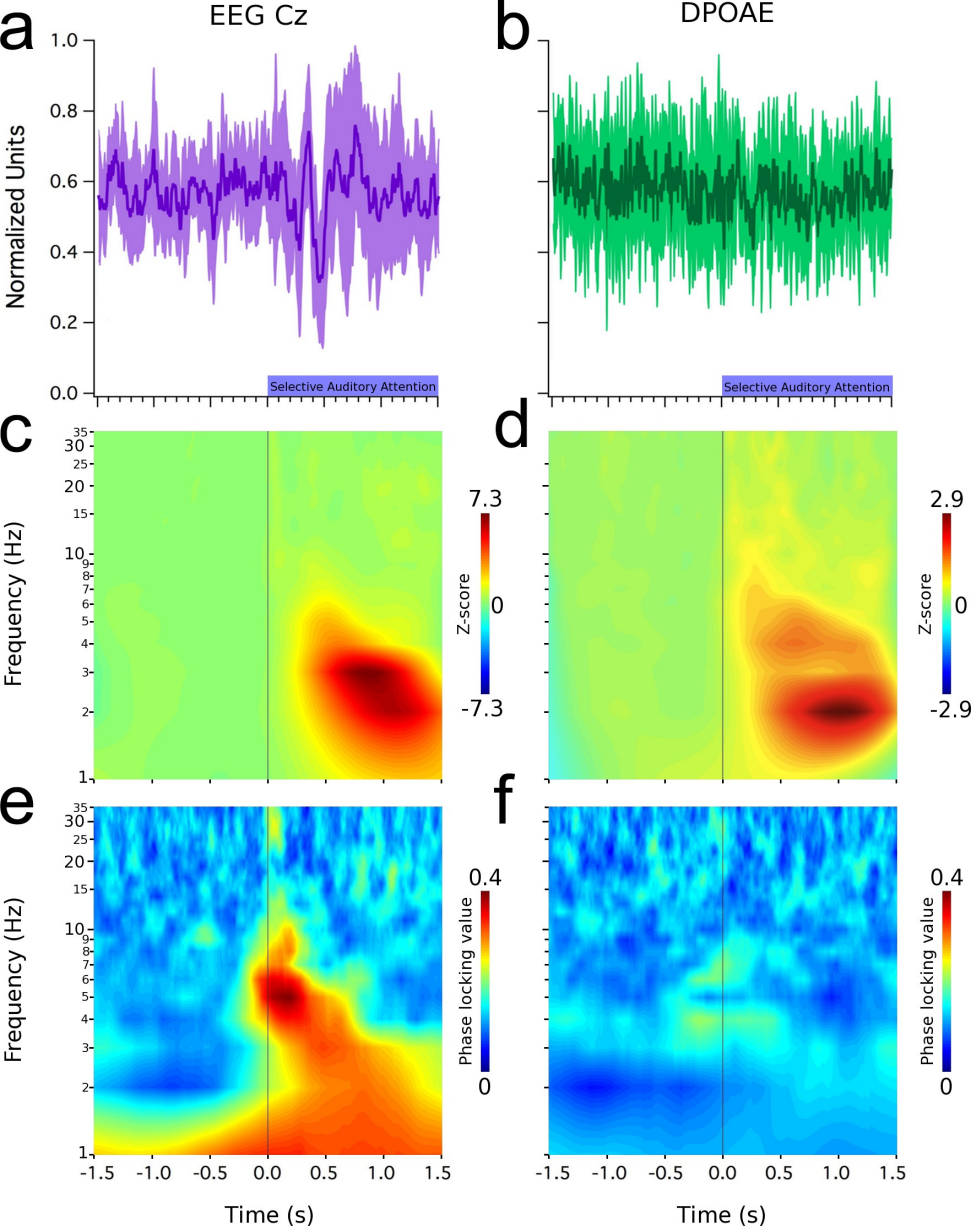
Low-frequency oscillations (< 10 Hz) in EEG (Cz electrode) and in the cochlea (DPOAE) during selective auditory attention. Time and frequency graphs represent grand average data. Left column shows: (a) evoked oscillations at Cz electrode (mean latencies: positive peak: 297 ± 6 ms; negative peak: 420 ± 9 ms), (c) time spectrum and (e) an increased phase locking value of these brain oscillations. Right column shows: (b) a lack of evoked effects in the amplitude of DPOAEs, (d) low frequency induced DPOAE amplitude oscillations observed between 1 and 7 Hz. (f) Notice the presence of small regions of DPAOE phase locking around 0 ms in cochlear oscillations. Shaded purple and green areas in (a) and (b) represent data dispersion (± 1 standard deviation).

During the period of auditory selective attention, in which subjects had high expectancy for a silence gap embedded in the continuous DPOAE-evoking primary tones (f1 and f2), an evoked potential appeared in the grand average of the EEG signal at Cz (Fig 2A), while in the same period a subtle non-significant reduction was observed in the DPOAE signal (Fig 2B). During this period, we also found the presence of low frequency oscillations (<10 Hz) in the brain (Fig 2C, EEG) and cochlear receptor (Fig 2D, DPOAE). EEG Cz oscillations were phase-locked to the onset of the auditory attention period (Fig 2E), while cochlear oscillations had a small region of phase locking at around 0 ms (Fig 2F). These increases in EEG and DPOAEs phase locking values (PLV) were above two and three standard deviations from the PLV baseline (S3 Fig).

**Fig 3:**
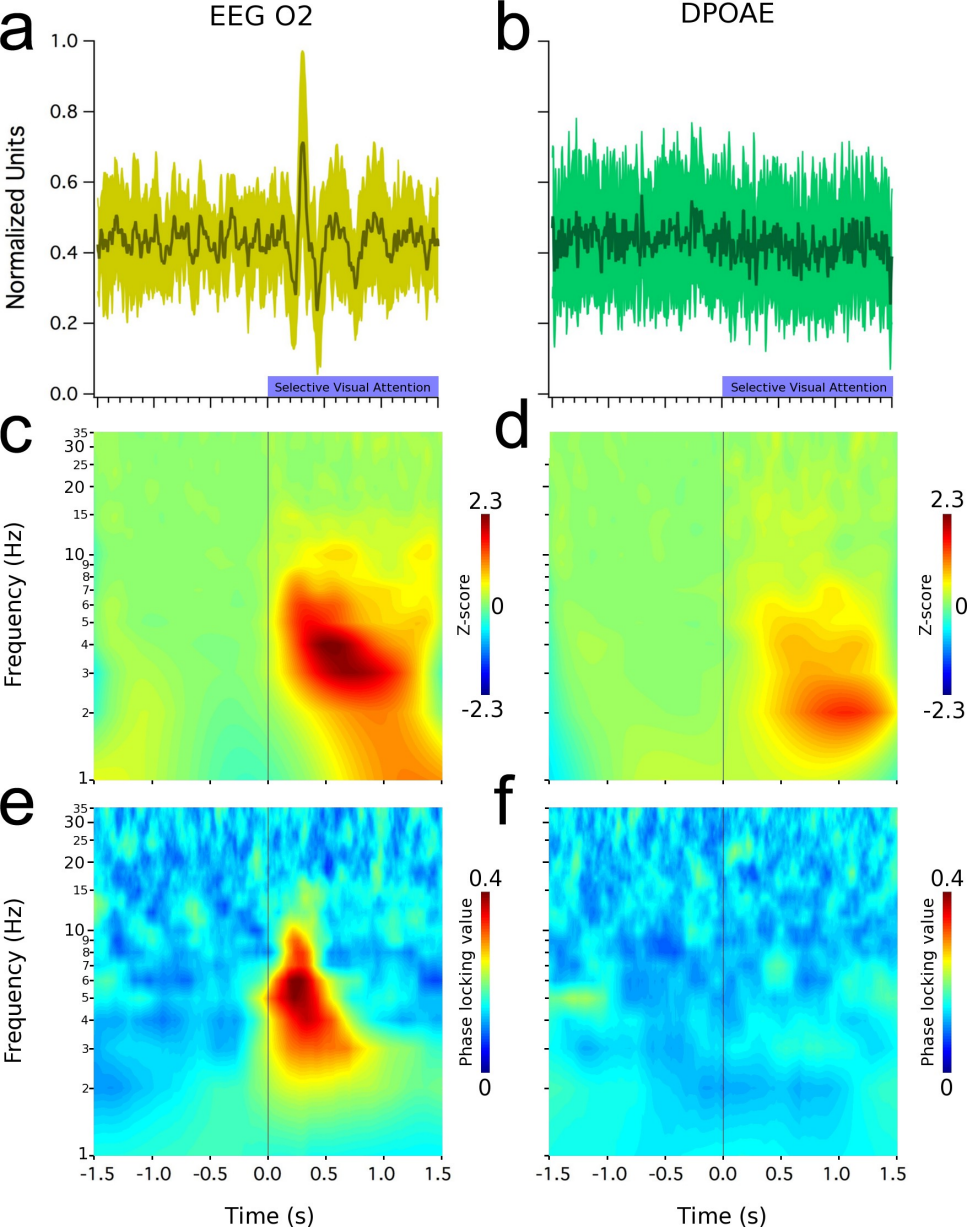
Low-frequency oscillations (< 10 Hz) in EEG (O2 electrode) and in the cochlea (DPOAE) during selective visual attention. All graphs represent grand average data. Left column shows (a) a visual evoked potential recorded from the right occipital cortex (mean latency: 340 ± 7 ms), (c) time spectrum and (e) increased phase locking values of these brain oscillations. Right column shows (b) a lack of evoked effects in DPOAE amplitudes, while (d) low frequency induced oscillations can be observed between 1 and 7 Hz. (f) Notice the presence of a small region of phase lock value increase at around -1200 ms, while in the rest of the graph there is no phase locking in cochlear oscillations. Shaded green areas in (a) and (b) represent data dispersion (± 1 standard deviation).

Fig 3 shows grand average results for the case of visual selective attention. A visual evoked response was clearly seen in the occipital EEG channels (Fig 3A), while the averaged DPAOE signal showed no effect (Fig 3B). Similarly to the auditory attention trials, the frequency analyses of EEG and DPOAE signals yielded the presence of low-frequency (<10 Hz) oscillations at the brain and cochlear levels (Fig 3C and 3D). The PLV of these oscillations, show that the EEG signal recorded from the occipital cortex is synchronized to the onset of the visual attention period (Fig 3E), while a small region of DPAOE phase-locking was obtained at around -1300 ms (Fig 3F, S3 Fig).

**Fig 4:**
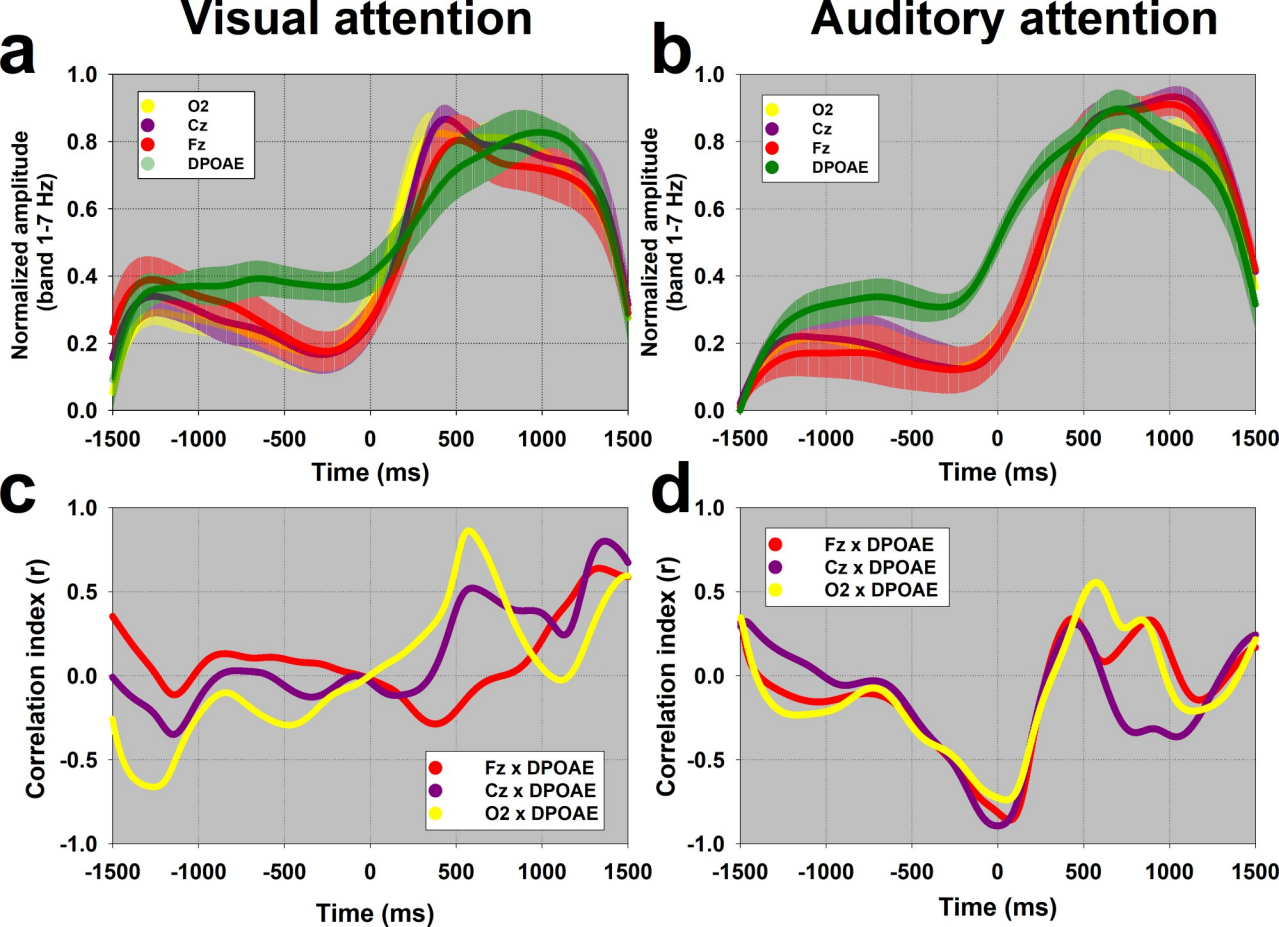
Temporal course and correlations of the amplitude of 1–7 Hz frequency band between EEG and DPAOE channels. The grand averages of the temporal course of the amplitude of the frequency band between 1 Hz and 7 Hz are shown in panels (a) and (b) for the visual and auditory attention tasks respectively (data shown as mean ± SEM). Notice that the DPAOE increment in the auditory attention task precedes those of EEG channels. The EEG and DPOAE curves shown in panel 4a,b were used to compute the half-time (50%) of the maximum oscillation amplitudes. Panels 4c and 4d show the temporal course of the Pearson correlation value (r) between the amplitude of DPOAE and Cz, Fz and O2 channels. Notice that around 500 ms there are positive correlations above 0.8 between DPAOE and O2 in the visual task, while in the auditory attention task there are significant negative correlations between DPOAE and EEG channels (~ -0.8) near 0 ms.

In order to compare amplitudes and temporal dynamics of single trial EEG and cochlear oscillations, in the visual and auditory attention tasks, the amplitude of the frequency band between 1 and 7 Hz were normalized as z-scores for both types of attention. For cochlear oscillations, a significant reduction in amplitude was observed during periods of visual attention (0.97 ± 0.48 z, Fig 3D) compared with those of auditory attention (1.63 ± 1.15 z, Fig 2D) (Z_(20)_ = -2.089, p = 0.038, Mann-Whitney). Fig 4A and 4B show the temporal course of the normalized amplitude of the 1–7 Hz frequency band in EEG and DPAOE channels during visual and auditory attention. There were increases in the amplitude of this frequency band at the brain and cochlear levels during periods of visual and auditory attention (from 0 to 1500 ms).

Next, we correlated the temporal course of the normalized amplitude of the 1–7 Hz frequency band between EEG (Cz, Fz and O2) and DPOAE signals in the visual and auditory attention tasks. Fig 4C shows significant positive correlations between the oscillation amplitudes of DPOAE and O2 EEG channels around 500 ms in the visual attention task, while Fig 4D shows significant negative correlations between the oscillation amplitudes of DPOAE and EEG channels (Cz, Fz and O2) around 0 ms in the auditory attention task.

**Fig 5:**
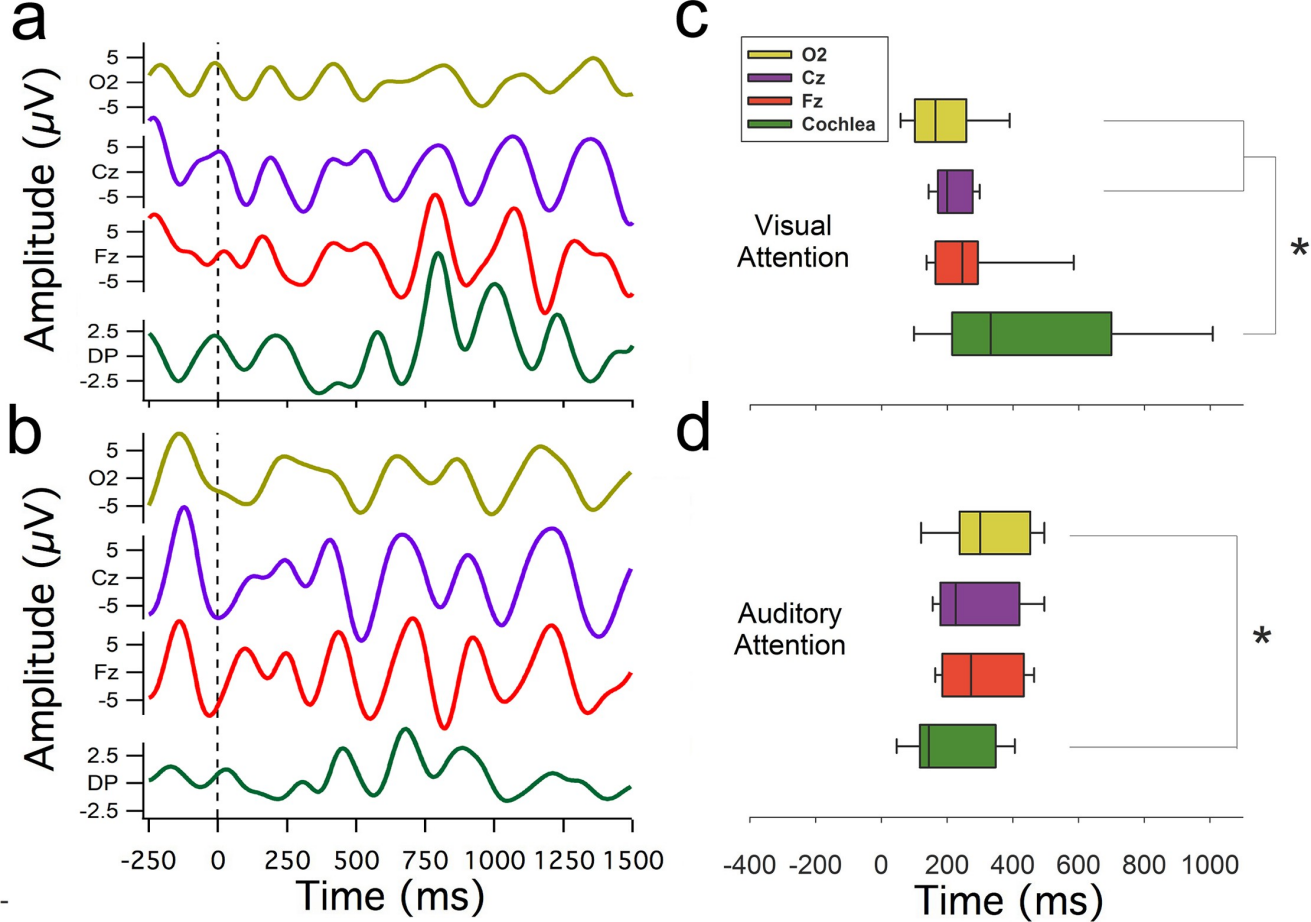
Temporal dynamics of brain and cochlear oscillatory activity. Examples of single trial EEG and DPOAE oscillations filtered between 1 and 7 Hz are shown in (a) visual attention and (b) auditory attention. Box plots in (c) and (d) represent, for the visual and auditory case respectively, the group distribution of the half times of the oscillatory peak activity in the 1–7 Hz frequency band of EEG recordings from O2 (yellow), Cz (purple), Fz (red) and cochlea (green, DPOAE signal). Asterisks denote a statistically significant difference of half-peak times (p<0.05, Mann-Whitney tests) between DPOAE amplitude and O2 for both modalities, and also with Cz in the visual attention case. Notice the wider distribution of half times in DPOAE oscillations (green boxes) during visual attention as compared to auditory attention.

From the curves shown in Fig 4A and 4B, we calculated the half-time necessary to obtain 50% of the maximum normalized amplitude of EEG and DPOAE oscillations (1–7 Hz, single trial examples shown in Fig 5A and 5B) during visual and auditory attention tasks. In the visual attention task, the half-time of the maximum amplitude of occipital cortex oscillations (median: 163 ms, interquartile range (IQR):101–257 ms) preceded DPOAE oscillations (median: 332 ms, IQR: 214–699 ms) (Fig 5C), while during auditory attention, this order was inverted as the cochlear amplifier oscillations (median 144 ms, IQR: 117–347 ms) preceded occipital cortex oscillations (median 300 ms, IQR: 238–453 ms) (Fig 5D). In addition, the half-times of DPOAE oscillations in the auditory attention task (median 144 ms) were significantly smaller than those of DPAOE oscillations in the visual condition (median 332 ms) (Z_(20)_ = -2.089, p = 0.038, Mann-Whitney).

## Discussion

In the present study, we report the presence of low-frequency (<10 Hz) EEG and DPOAE oscillations during a crossmodal task that shifts between visual and auditory selective attention. Many authors consider primary sensory cortices as the earliest brain regions that could be affected by the oscillatory mechanisms in the attentional network. The present findings expand the framework of these mechanisms by adding the cochlear receptor as the most peripheral structure modulated by the attentional oscillatory network.

It is important to keep in mind that otoacoustic emissions are sounds–pressure waves–emitted by the inner ear that can be measured with a sensitive microphone positioned at the external ear canal [20]. They are thought to reflect the electro-motility of outer hair cells of the cochlear receptor, which is the proposed cellular mechanism for cochlear amplification [19,21,22]. The frequency band (<10 Hz) of the oscillations modulating the amplitude of DPOAEs (2f1-f2) that we found is located below the human audible range (which goes from 20 to 20,000 Hz), and therefore, these low-frequency oscillations can be considered as infrasound waves [23].

Whether attention modulates the cochlear receptor has remained controversial for many years [3,4]. A number of works have found top-down effects of visual attention at the cochlear amplifier measuring otoacoustic emission amplitudes [8,24–26], however, other authors have failed to find them [3,4,9]. In this study, we also explored frequency specific oscillatory mechanisms for attentional selection at the cochlear level. Remarkably, although we did not find a modulation of the mean amplitude of evoked DPOAEs by selective attention (Figs 2B and 3B), we did find low-frequency oscillations in DPOAE amplitudes during selective attention to visual and auditory stimuli.

A previous study [8] attempted to investigate the modulatory effects of selective attention on EEG and DPOAE recordings. Although the authors found a significant reduction of the power of alpha oscillations at the occipital cortex and a significant decrease of DPOAE amplitudes during visual attention, they did not find any statistical correlation between both measures. We found DPOAE amplitude oscillations in a frequency band similar to that of EEG oscillations (1–7 Hz) during auditory (Fig 2) and visual attention (Fig 3). Moreover, the amplitude of EEG and DPOAE oscillations were significantly correlated in different periods of the visual and auditory tasks (Fig 4C and 4D). Importantly, the temporal order of the electrical brain oscillations at the occipital cortex and the mechanical oscillations at the cochlear receptor were inverted depending on the attended modality (Fig 4A and 4B; Fig 5C and 5D). When the subjects were focused on the acoustic stimuli, then cochlear oscillations led EEG waves, while the opposite order was found for visual attention. Despite the inversion of the temporal order between occipital cortex and DPOAE oscillations produced by switching between auditory and visual attention, these findings do not suppose causality between brain and inner ear oscillations.

The low-frequency EEG oscillations that we observed in our tasks (1–7 Hz) can be classified as delta and theta oscillations [12]. Regarding theta oscillations, it has been theorized that in cognitive tasks they emerge in the frontal cortex [27] and serve as a time reference for the dynamic assembly of different neural populations (e.g. hippocampus), by increasing and decreasing the firing rate probability of single neurons subjected to the extracellular local field potentials induced by global cortical oscillations [28,29]. In the context of attention, theta oscillations have been found in cross-modal paradigms involving a switch between visual and auditory attention [30,31]. These authors showed that theta activity allows fronto-parietal top-down modulation of visual and auditory cortices during cross-modal attention. In our work we extend the oscillatory network of top-down attention towards the cochlear receptor by showing that occipital EEG low-frequency oscillatory activity precedes mechanical oscillations in the cochlear amplifier during visual attention. Remarkably, in the case of auditory attention, we found significant correlations between EEG and DPOAE oscillations that were mainly observed in the period of the attentional switch from the visual to the auditory modality (around 0 ms, Fig 4D). In this sense, we speculate that these DPOAE low-frequency oscillations are part of a larger network of theta oscillations related to cross-modal attentional switching.

Regarding the neuroanatomical pathways that may be responsible for the oscillatory cochlear effects observed during selective attention, we propose that the descending pathways from the auditory cortex to the cochlear receptor that comprise the auditory efferent system [32,33], are the most probable neural pathways that could explain the modulation of low-frequency oscillatory amplitude changes of DPOAEs. Evidences in bats [34] and chinchillas [35, 36] have demonstrated that the inactivation and electrical stimulation of the auditory cortex can modulate cochlear responses in a frequency specific manner. In addition, Aedo et al. [37] showed in the alpha-9 nicotinic receptor knock-out mice that these corticofugal effects are produced through the medial olivocochlear system.

During visual attention cochlear oscillations have a significant temporal jitter as compared with EEG oscillations (see boxplot IQRs in Fig 5C), which might reflect an active process to reduce the peripheral entrainment of auditory stimuli during visual attention. This possible mechanism would be in agreement with studies showing that low-frequency oscillations can modify the mechanical sensitivity of the cochlear receptor [18,38]. On the other hand, during auditory attention, cochlear oscillations precede EEG low-frequency oscillations, and less jitter is observed (Figs 4B and 5D), thus allowing entrainment of cochlear responses to auditory stimuli. The latter proposal would be in line with a general mechanism of oscillatory entrainment during attention to the corresponding relevant stimulus [14,39].

In summary, we found EEG and cochlear amplifier infrasonic oscillations during selective attention to visual and auditory stimuli. Moreover, the attentional switch between visual and auditory attention modulates the amplitude and the temporal order of brain and inner ear oscillations. These results extend the role of oscillatory activity in the nervous system during cognition to the receptor level.

## Materials and methods

### Ethics statement

This study was approved by the ethics committee at the Clinical Hospital of the Universidad de Chile, permission number: OAIC 016/20042016. All procedures were conducted in accordance to this protocol and to national regulations.

### Participants

Fourteen right-handed volunteers participated in our experiments (four females, mean age 24.2 ± 4.0 (SD, standard deviation)). All volunteers provided written consent and did not have any hearing or neurological impairments. Because of the strict procedures to remove EEG and DPOAE artifacts (see below), we excluded electrophysiological data from one subject from the visual attention task, and five subjects from the auditory attention task.

### General experimental procedures

All procedures were carried out in an acoustically isolated room designed for audiological and electrophysiological evaluations within the Clinical Hospital of the Universidad de Chile. Electroencephalographic signals (32-channel EEG, Tucker Davis Technologies) and continuous DPOAE dynamics were recorded simultaneously during an attention task that switches between visual and auditory modalities (Fig 1). A multifunction data acquisition board (National Instruments, NI6321) and a Tucker-Davis Technologies multiprocessor (model RZ6) housed in a desktop computer controlled the experiment through custom made software written in C (Labwindows/CVI 2009) and system 3 languages from Tucker-Davis Technologies.

Before positioning any measuring devices on the subjects, external ear canals were inspected for earwax, and if present, was removed. We then set up the EEG recording, followed by fitting the insert earphones and microphone for DPOAEs recording. We calibrated the sound level, and measured DPOAEs at different frequencies (between 0.5 and 4 kHz) in order to choose the primary tones parameters that elicited the cleanest DPOAE signal. Subsequently, we gave instructions to the subjects, verifying their understanding and execution through supervised training blocks, we then proceeded with the main experiment.

### DPOAE

During the experimental protocol, primary tones f1 and f2 were presented to the right ear continuously (ER-2, Etymotic Research) in order to elicit 2f1-f2 DPOAEs, which were recorded during approximately 8 minutes by a microphone (ER-10C, Etymotic Research) sealed to the external right ear canal. Before the experimental protocol, a set of nine pairs of tones with corresponding frequencies (f1 and f2) and intensities (L1 and L2) were generated using L2 fixed at 55 dB SPL and F2 with frequencies between 1 and 5 kHz. Calibration of each frequency was completed separately for both phones (phone A dealing with f1 and phone B with f2), by playing, adjusting, and replaying long tones (4,000 ms) until reaching a 50 dB constant sound pressure level (SPL). We then determined, for each subject, which pair of tones produced the largest DPOAE signals, based on 20 presentations of each tone pair (stimuli lasted for 1,000 ms and had an inter-stimulus interval of 500 ms). This was judged and manually selected by the authors, based on the graphical inspection of the spectrum of the averaged DPOAE signal, and on three parameters for each pair of f1 and f2 tones: (a) absolute peak amplitude at DPOAE frequency 2f1-f2, (b) amplitude of surrounding noise (for frequency band ± 10 Hz), and (c) standard deviation of surrounding noise. With these parameters, we calculated the difference between DPOAE amplitude and surrounding noise (a—b), and the difference between this difference and the standard deviation of the surrounding noise ([a—b]—c). The main experiment was performed with the tone parameters selected in this step; the frequency of the tones used for each subject can be seen in S1 Fig.

### EEG

We recorded a total of 32 EEG (referenced to the right earlobe) and two electro-oculogram (EOG) channels (for vertical and horizontal eye movements) that were preamplified and digitized by battery powered Tucker-Davis Technologies devices (PZ3 for EEG and RA4PA for EOG). Ring shaped Ag/AgCl electrodes were positioned in an elastic headcap (size 56 or 58, EasyCap, Germany) that was secured with velcro under the chin area. EEG electrode positions complied with the 10–20 EEG standard system. Ground electrode was positioned on Fz. Scalp contacts were cleaned with alcohol and electroconductive gel was applied to keep impedances < 5 kΩ. Data were digitally filtered using a band pass filter (0.1–100 Hz) and a notch filter at 50 Hz. The output of this filtered data was saved with a sampling rate of 1 kHz.

### Attention tasks

During the visual attention task, DPOAE eliciting tones (f1 and f2) served as distractors. There were no silence gaps during the complete window of analysis in the visual attention task (from -1500 up to 1500 ms). In contrast, during the auditory attention task the DPOAE eliciting tones (f1 and f2) had to be attended for detecting a brief gap of silence (5 ms squared cosine ramps and 2–4 ms of complete silence). The perceptual modality to be attended alternated after valid responses or after the end of response time windows, 100–1,000 ms from target. Subject responses were given with the right thumb through a custom-made push button. Volunteers completed four experimental blocks of 44 trials for each modality, following at least 1 training block (explained below). Each block had an approximated duration of eight minutes.

#### Visual task, stimuli and apparatus

The visual task started with a passive (no attention) pseudo-random period lasting between 2,000 and 2,500 ms. During this period, subjects were instructed to maintain fixation at the center of a single-handed clock 4° in diameter. The clock hand revolves clockwise at 1 Hz, passing through 100 tick marks. To accomplish synchronization between the custom software and the high refresh rate (100 Hz) monitor (Samsung LED 23" 3D S23A700D), a time counter of the National Instruments multifunction board was configured to trigger screen refresh at the same 100 Hz. This ensures a smooth and coherent motion perception. Randomly, at some point during the 2,000–2,500 ms passive period, a visual cue appears as a change in color of the external rim of the clock, indicating the period of visual selective attention (shown in blue on Fig 1). In the visual attention task, time “0” ms corresponds to the onset of the visual cue (change of the external rim of the clock). Individuals had to report as precisely as possible the clock hand position at the time of visual cue offset occurring 1,500–2,500 ms from its onset. This task was adapted from a similar version implemented by other investigators [17]. To determine at what time the subjects thought that the cue offset occurred, the button had to remain pressed, inverting the rotation of the clock hand (from clockwise to counter-clockwise) and slowing down to 0.33 Hz, eventually passing over the target position, where the button had to be released to set the response. No feedback was provided to the subjects regarding performance. Immediately following button release, the task switches to auditory attention, and the clock hand no longer moves but jumps to random positions without coherent motion.

#### Auditory task, stimuli and apparatus

When the task switches to the auditory selective attention, subjects must react by pressing the button when they detect a brief silence gap that interrupts the continuous DPOAE-eliciting tones. They must focus on the auditory domain while ignoring random jumps in the position of the clock hand. In the auditory attention task, time “0” ms corresponds to the attentional switch (behavioral response of the visual attention task) and no silence gaps were presented between 0 and 1,500 ms. Silence gaps occur randomly between 1,500–2,500 ms. Following the appearance of the silence gap, a response period of 1,000 ms was given to react upon its detection. The task switches to the initial passive visual period if the response window ends (omitted trial), or immediately after correct detections (button press between 100–1,000 ms from gap onset), which turns the random pattern of the clock hand again into clockwise, coherent rotation at 1 cycle per second.

Acoustic stimuli generation and recordings of DPOAE were performed by RZ6 multiprocessor at a sampling rate of 48 kHz. Gaps in sound were digitally generated with squared cosine rise/fall ramps of 5 ms to avoid click-type acoustic artifacts on the onset and offset of the gap. Etymotic Research equipment (ER-2 and ER10-C) specifically designed for human recording of DPOAEs was used to deliver sounds and record otoacoustic emissions via three physical channels (two output phones for each primary tone, and one microphone) gently sealed to the external ear canal with foam earplugs.

#### Training blocks

After we confirmed that recordings were robust (low impedance of EEG signals and clear DPOAEs respect to surrounding noise), we explained the tasks instructions to the subjects, and let them practice with short blocks (11 trials) in which the visual target (instantaneous clock hand position at the moment of cue offset) remained visible (as a thin line) until the response was set. We also verified that the subjects heard the gaps in sound stimuli during the alternated auditory tasks, and if not, or if the visual task was not understood, another training block was presented with longer (easier) silence gaps if necessary.

### DPOAE channel

For analyses of the DPOAE channel, we took a novel approach: the DPOAE amplitude was extracted from the raw microphone signal and transformed into a ‘virtual’ channel that was added to the set of electrophysiological EEG/EOG, all down-sampled to a sampling rate of 256 Hz. This allowed us to study amplitude oscillations of the cochlear amplifier in the same band of frequencies (1–35 Hz) as that of cognitive tasks that are commonly evaluated in EEG research. At the technical level, this method provided the benefits of the EEG analysis techniques provided in the free software ELAN [40], for time and frequency domain measures. Two methods for measuring DPOAE amplitudes were implemented by custom code in Igor 6 (Wavemetrics). A Fourier-based method divided the signal into 16.7 ms (1/60 Hz) running windows and applied fast Fourier transform (FFT) to each. The actual length of windows was adjusted for each case in order to be a multiple of the period of the DPOAE frequency 2f1-f2 selected for each subject. The other, a Hilbert-based method, implied first band-passing the signal through a filter with strong attenuation (>110 dB) at the frequency of the f1 primary tone, whose amplitude was typically 60–70 dB greater than the DPOAE amplitude. Having the band-pass signal and its Hilbert transformation, the envelope, representing the amplitude of the DPOAE band, was calculated. The two methods yielded similar results, validating our method of continuous DPOAE amplitude extraction.

### Pre-processing of the main experiment

EEG and DPOAE artifacts were rejected by visual inspection of single trials. Rejection according to DPOAE amplitude was completed by visual inspection of the complete time series from each recording block. Cursors were positioned graphically to delimit the range of valid amplitude values (see S2 Fig for example of DPOAE artifact removal). The unrejected DPOAE trials were then inspected one by one in the ELAN software with a selected group of channels: vertical EOG, horizontal EOG, Fourier-based DPOAE, Hilbert-based DPOAE, and then EEG channels Fz, F3, Fc1, Cz, Fc2, F4, P3, O1, Pz, O2, P4). Trials presenting with any kind of artifact were rejected by this procedure. The remaining EEG and DPOAE trials were then analyzed with ELAN tools in the time and frequency domains.

### Data analyses

Time and frequency averages of the EEG and DPAOE channels across trials were locked to either the onset of the visual cue (0 ms in visual attention) that started the period of focused visual attention, or to the attentional switch that initiates the auditory attention task triggered by the behavioral response of the previous visual task (0 ms in auditory attention). For time and frequency analysis purposes, we used time windows of ± 1,500 ms aligned to the onset of visual or auditory attention periods.

We analyzed frequencies between 1 and 35 Hz, in steps of 1 Hz, with Morlet wavelets having m ratio equal to seven. First, we calculated the average of spectral z-scores. For each subject and channel, the spectrum of the single trials was obtained with the wavelet method, and frequency specific z-scores were obtained based on each trial baseline (-1,500 to 0 ms). In other words, for each trial and frequency value, the mean and standard deviation of the baseline period was calculated, and the whole spectrogram represented in z-score. Finally, these spectrograms were averaged for each subject, and then across subjects. We then measured the inter-trial-phase locking values, which measures the consistency of phase alignment across trials. Only the phase of each frequency component was considered and not its amplitude. Values are bounded between 0 and 1, from null to complete phase synchrony across trials.

To study the time relationship between the emergence of cortical and cochlear oscillations revealed by the average of spectral z-scores, we measured the time points where 50% of the maximum oscillatory power was achieved in the band between 1 and 7 Hz, for Cz, Fz, O2 and the DPOAE-amplitude channels and were compared for the visual and auditory conditions. The EEG channels: Cz, Fz, and 02 were chosen as a measure of the auditory cortex, prefrontal cortex and visual cortex respectively. The curves used to calculate the half-time of the maximum amplitude of EEG and DPOAE oscillations are plotted in Fig 4A and 4B, while the half-time value dispersion are box-plotted in Fig 5C and 5D. The statistical significance of amplitude and temporal changes between EEG and DPAOE channels were evaluated with the Mann-Whitney test, while temporal correlations between EEG and DPOAE channels were evaluated by Pearson tests (using p<0.05 as significant).

## Supporting information

S1 Fig DPOAE band spectra of individual subjects. FFT of the band-pass filtered microphone signal of each subject prior to Hilbert transform. Each subplot corresponds to one block of one subject. Filters were centered at DPOAE frequency (2f1-f2), and had a flat frequency response with no attenuation in a ± 50 Hz vicinity. Amplitude is shown in attenuation dB relative to the DPOAE peak amplitude.
(TIF)

S2 Fig DPOAE-based artifact rejection. Representative example of the time course of DPOAE amplitude in one block of a subject, illustrating the first rejection stage based solely on DPOAEs. Horizontal dashed lines were manually positioned to define rejection limits (shown by “A” and “B”). Only trials that were not rejected by this process were further scrutinized in the following EEG artifact rejection stage.
(TIF)

S3 Fig Phase locking values for both attentional tasks. Colors represent data regions deviating in the negative direction more than two (blue), or in the positive direction more than two (orange) and three (red) standard deviations from the average. (a): PLV of the DPOAE amplitude channel for the auditory attention task. (b) PLV of the DPOAE amplitude channel for the visual attention task. (c) PLV of the Cz EEG channel for auditory attention. (d) PLV of the O2 EEG channel for the visual task. White areas represent data in the range between average ± 2 standard deviations (Aud: auditory attention; Vis: visual attention).
(TIF)
