# Supplementary figures and images for: Oscillatory infrasonic modulation of the cochlear amplifier by selective attention
Source: PLoS One. 2019 Jan 7;14(1):e0208939. doi: 10.1371/journal.pone.0208939 (PMC6322828; doi:10.1371/journal.pone.0208939)

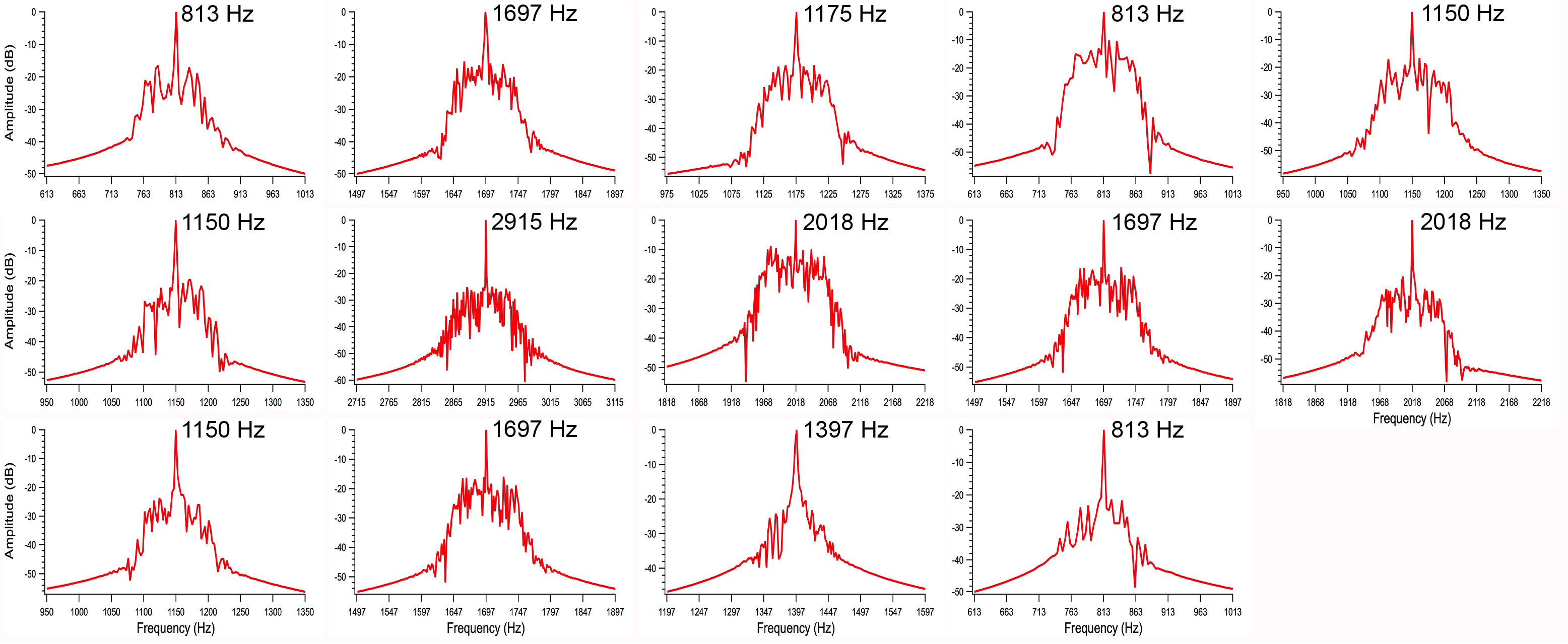

Supplement: S1 Fig — FFT of the band-pass filtered microphone signal of each subject prior to Hilbert transform. Each subplot corresponds to one block of one subject. Filters were centered at DPOAE frequency (2f1-f2), and had a flat frequency response with no attenuation in a ± 50 Hz vicinity. Amplitude is shown in attenuation dB relative to the DPOAE peak amplitude. (TIF) [file pone.0208939.s001.tif]

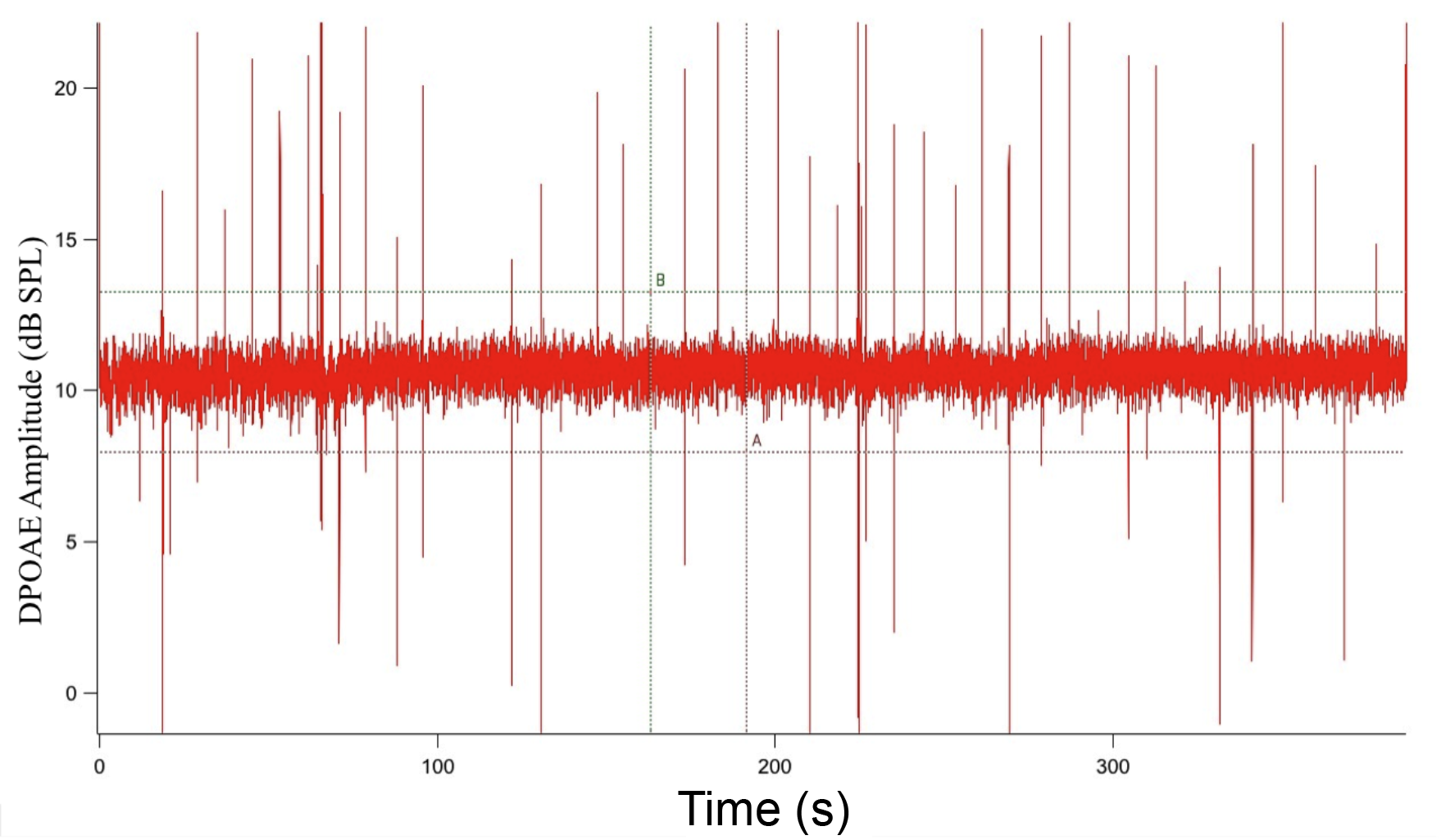

Supplement: S2 Fig — Representative example of the time course of DPOAE amplitude in one block of a subject, illustrating the first rejection stage based solely on DPOAEs. Horizontal dashed lines were manually positioned to define rejection limits (shown by “A” and “B”). Only trials that were not rejected by this process were further scrutinized in the following EEG artifact rejection stage. (TIF) [file pone.0208939.s002.tif]

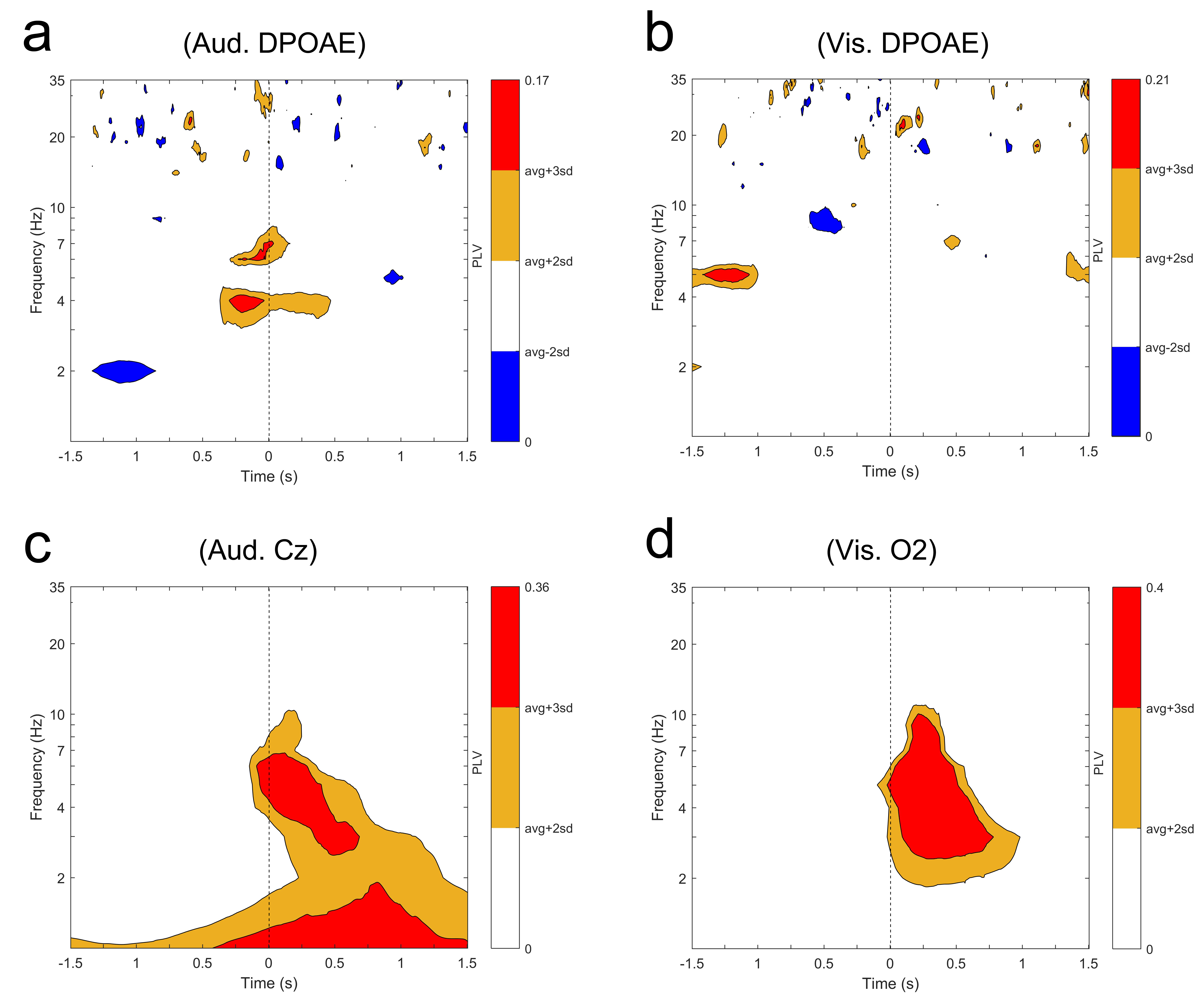

Supplement: S3 Fig — Colors represent data regions deviating in the negative direction more than two (blue), or in the positive direction more than two (orange) and three (red) standard deviations from the average. (a): PLV of the DPOAE amplitude channel for the auditory attention task. (b) PLV of the DPOAE amplitude channel for the visual attention task. (c) PLV of the Cz EEG channel for auditory attention. (d) PLV of the O2 EEG channel for the visual task. White areas represent data in the range between average ± 2 standard deviations (Aud: auditory attention; Vis: visual attention). (TIF) [file pone.0208939.s003.tif]
